# Update on the “Choosing Wisely” initiative in infectious diseases in Germany

**DOI:** 10.1007/s15010-020-01400-z

**Published:** 2020-03-10

**Authors:** Rika Draenert, Norma Jung, the Choosing Wisely (Klug entscheiden) DGI Working Group, Marylyn M. Addo, Reinhard Berner, Markus Bickel, Johannes Bogner, Oliver Cornely, Katja de With, Gerd Fätkenheuer, Stefan Hagel, Frank Hanses, Thomas Harrer, Pia Hartmann, Susanne Herold, Stefan Hippenstiel, Winfried Kern, Stephan Klauke, Hartwig Klinker, Clara Lehmann, Sebastian Lemmen, Christoph Lübbert, Mathias W. R. Pletz, Siegbert Rieg, Bernhard R. Ruf, Jan Rupp, Bernd Salzberger, Hortense Slevogt, Hans-Jürgen Stellbrink, Christoph Stephan, Norbert Suttorp, Andrew Ullmann, Martin Witzenrath, Oliver Witzke

**Affiliations:** 1 LMU Klinikum, Stabsstelle Antibiotic Stewardship, Ludwig-Maximilians-University, Munich, Germany; 2 Division of Infectious Diseases, Department of Internal Medicine, University of Cologne, Cologne, Germany

**Keywords:** Choosing Wisely, Infectious diseases, Vaccination, Meningitis, Blood cultures

## Abstract

**Purpose:**

The Choosing Wisely^®^ initiative is an international campaign addressing over- and underuse of diagnostic and therapeutic measures in infectious diseases among others. Since 2016, the German Society for Infectious Diseases (DGI) has constantly designed new items in this regard. Here we report the most recent recommendations.

**Methods:**

The recommendations of the DGI are part of the “Klug entscheiden” initiative of the German Society of Internal Medicine (DGIM). Topics for the new items were suggested by members of the DGI, checked for scientific evidence and consented within the DGI and the DGIM before publication.

**Results:**

The new recommendations are: (1) individuals with immune-suppression, advanced liver cirrhosis or renal insufficiency should receive a dual pneumococcal vaccination. (2) In case of positive blood cultures with *Candida *spp*.* thorough diagnostics and treatment should be initiated. (3) In case of suspected meningitis, adult patients should receive dexamethasone and antibiotics immediately after venipuncture for blood cultures and before potential imaging. (4) In case of suspected meningitis a CT scan before lumbar puncture should not be ordered—except for symptoms indicating high CSF pressure or focal brain pathology or in cases of severe immune-suppression. (5) In patients with suspected severe infections, a minimum of two pairs of blood cultures should be drawn using separate venipunctures prior to antibiotic therapy—regardless of body temperature. There is no need of a minimum time interval in between the blood draws.

**Conclusion:**

Applying these new Choosing Wisely^®^ recommendations will increase patient safety and the value of health care.

## Introduction

The Choosing Wisely^®^ campaign was first initiated in the USA in 2012. Soon other countries followed, including Germany. Since 2016, 125 recommendations have been published in the section of infectious diseases as well as other specialties of Internal Medicine in Germany [1]. However, a further development seems pertinent as there are still many areas untouched so far.

Vaccination acceptance is a main concern of the German Society of Infectious Diseases (DGI), especially in times of waning vaccination rates due to increasing scepticism to vaccination globally. Therefore, vaccines were included in the recommendations of the DGI from the beginning. Besides a rational application of antibiotics and other anti-infectives, fungal diseases are of utmost importance as their incidence is increasing, and they mostly occur in severely ill and vulnerable patients leading to a high mortality rate. Difficulties to deal with comprise the diagnosis as well as treatment conditions including the dosage and serum levels of antifungals as well as drug–drug interactions. Another area of concern to the DGIM is the emergency department. Here the time constraint due to high patient numbers and seriously ill individuals has to be taken into account. Last but not least diagnostics are a most relevant issue in infectious diseases, providing the opportunity of characterizing the causative agent in detail which is crucial for the optimal antimicrobial treatment. On the other hand, overuse of diagnostics can delay the urgently needed application of antibiotics. All this is reflected in the new recommendations—positive and negative—presented here.

## Methods

The Choosing Wisely^®^ recommendations were created as described previously [2]. Shortly, committee members of the DGI suggested important issues suitable for new Choosing Wisely^®^ items which were checked for scientific evidence and overlap to older recommendations. Two general and three emergency room recommendations were finally chosen. After the out-writing of the recommendations, they were discussed and consented by the Choosing Wisely^®^ commission of the DGI and the DGIM. The recommendations concerning bacterial meningitis were jointly agreed upon by the German Society of Neurology (DGN).

## Results

### General recommendations

*Individuals with immune-suppression, advanced liver cirrhosis or renal insufficiency should receive a dual pneumococcal vaccination*.

Patients with immune-suppression are exposed to a significantly higher risk of severe pneumococcal infections, depending on the kind of immune suppression, with the highest risk after splenectomy. Additional conditions which are associated with immune-suppression such as chronic liver cirrhosis and renal insufficiency or certain anatomical risks (e.g. cerebrospinal fluid leaks or cochlea implants) are predisposing factors for pneumococcal infections. Since 2016, the “STIKO” (the German Commission on Vaccination) recommends a dual vaccination with the 13-valent conjugate vaccine (PCV13) followed by the 23-valent polysaccharide vaccine (PPSV23) 6–12 months later in these patient groups. If the polysaccharide vaccine has been given previously, the conjugate vaccine should not be used before the course of 1 year, to achieve a better immune response. There are specific vaccination schedules for patients after stem cell transplantation. Compared to the polysaccharide inoculum, the conjugate vaccine induces memory cells. Especially for HIV infected individuals, a protective effect of the conjugate inoculum was shown whereas this could not be demonstrated for the polysaccharide vaccine. The 13-valent conjugate vaccine, however, comprises only about 30% of pneumococcal infections in adults, in contrast the 23-valent polysaccharide inoculum about 60–70%. Therefore, the sequential vaccination with both inocula conveys the best protection against pneumococcal infections at the moment [3–8].

*In case of positive blood cultures with* *Candida spp. thorough diagnostics and treatment should be initiated.*

*Candida *spp*.* are a frequent cause of bloodstream infections und are associated with a mortality of 30–40%. Even a single positive blood culture with *Candida *spp*.* is relevant. For the medical treatment, echinocandins are primarily used due to their superior efficacy and their favourable profile regarding adverse events. Fluconazole is not a safe first-line regimen. Treatment duration is at least 14 days starting with the first negative blood culture which should be performed on close follow-up after the first positive culture. After documented clearance of *Candida *spp*.* from the blood stream, the therapy can be switched to fluconazole or voriconazole if the causative organism was tested susceptible and the clinical status is stable. In certain cases, oral administration of antifungals is also possible. One of the key factors in the management of *Candida *spp*.* bloodstream infections is the identification of the correct focus. Intravascular catheters should be removed immediately. If the *Candida *spp*.* bloodstream infection persists for more than 4 days echocardiography should be performed to rule out *Candida* endocarditis [9–11].

### Recommendations for the emergency department

*In case of suspected meningitis, adult patients should receive dexamethasone and antibiotics immediately after venipuncture for blood cultures and before potential imaging*.

Bacterial meningitis is a severe infection with high morbidity and mortality. Every delay in treatment is associated with a worse prognosis. Antibiotics—directly after dexamethasone—should be given as soon as possible after presentation to the emergency department.

Typical symptoms are fever, headache, nuchal rigidity, altered mental status and massive reduction of well-being; the absence of single symptoms including nuchal rigidity, however, does not exclude bacterial meningitis. In suspected meningitis, blood draw and lumbar puncture should be performed immediately. In case of a delay of lumbar puncture (e.g. indication for cranial CT before the procedure), antibiotics should be given before lumbar puncture is performed [12–16].

*In case of suspected meningitis a CT scan before lumbar puncture should not be ordered—except for symptoms indicating high cerebrospinal fluid (CSF) pressure or focal brain pathology or in cases of severe immuno-suppression*.

Within the diagnostics for bacterial meningitis, a lumbar puncture is of paramount importance in order to confirm the diagnosis, to identify the causative agent and its resistance profile. It is mandatory for an optimal antibiotic therapy. A cranial CT scan before lumbar puncture can delay the application of antibiotics and dexamethasone and is performed too often in daily clinical practice which is not according to current guidelines. Indications for a cranial CT scan preceding lumbar puncture are: (1) focal neurological symptoms, (2) first-time epileptic seizures, (3) massive altered mental status (GCS < 10) or (4) severe immunosuppression.

Patients without one of the aforementioned criteria do not need a cranial CT scan because the detection of abnormalities leading to contraindication against lumbar puncture is highly unlikely.

Severe immunosuppression is defined—among others—as: severe innate immunodeficiency, CD4 cell count < 200/µl, status post allogeneic stem cell or organ transplantation, intensive immunosuppression with two or more drugs, corticosteroid intake of > 0.5 mg/kg/day (prednisolone equivalent) within the last 4 weeks or longer [16–24].

*In patients with suspected severe infections, a minimum of two pairs of blood cultures should be drawn using separate venipunctures prior to antibiotic therapy—regardless of body temperature. There is no necessity of a minimum time interval in between these blood draws.*

Severe infections (e.g. sepsis, septic shock, meningitis, pneumonia, endocarditis) requiring hospital admission are common diseases in emergency departments. For optimal treatment of these severe diseases, knowledge of the causative agent is essential. Bacteremia is frequently associated with a severe course of infection, e.g. concomitant bacteremia is found in about 40% of the cases in pneumococcal pneumonia. Therefore, blood cultures are an important diagnostic tool. Contrary to earlier assumptions, there is no correlation between rising fever and a high bacterial load in the blood. The sensitivity of blood culture diagnostics increases from 73 (one pair of blood cultures) to 90% in case that two pairs of blood cultures are drawn. In suspected endocarditis, three pairs of blood cultures should always be drawn. The time interval between the blood culture venipunctures is not important. However, blood cultures should be drawn at different venipuncture sites in order to be able to recognize contamination easily. The time point of venipuncture is rather secondary and it should not delay the urgent start of antimicrobial therapy [25–29].

## Discussion

Here, we report the advancement of the Choosing Wisely^®^ recommendations in the field of infectious diseases in Germany. Preventive measures such as vaccinations and the diagnostic and therapeutic management of *Candida *spp*.* bloodstream infections and meningitis present the focus of this round’s items.

The Choosing Wisely^®^ campaign was initiated in 2012 in the USA. Since then several countries have started their own campaigns. This initiative is not terminated but new recommendations are being developed continuously reflecting the medical need. Accordingly, the collection has been developed further during the past years in Germany, now also including special aspects in medicine, e.g. emergency medicine and vaccinations. This was done particularly due to the common assessment of the commission that many important questions had not been touched yet.

A point of debate within the Choosing Wisely^®^ campaign is a targeted distribution to suitable addressees. How can this be made known to a substantial proportion of doctors? The DGIM has approached this with publications in widely read journals in Germany, lectures and sessions on conferences and—just recently—e-learning modules using learning by case solving (https://www.klug-entscheiden.com/). However, additional multipliers are desirable in order to ascertain wide-spread publicity not only among physicians in the hospital and in the private practices but also directly among patients—the latter being an important principle of the Choosing Wisely^®^ campaign, namely encouraging the critical dialogue between patients and their physicians.

The augmentation of the Choosing Wisely^®^ collection leads to the question of acceptance. At the moment, the implementation of our recommendations is not assessed and therefore unclear in Germany. It would be desirable, however, to evaluate the proportion of physicians using the recommendations in their daily activities and the proportion of doctor-patient conversations regarding application of the recommendations in real-life medicine. Other countries, however, have started to address this question [30–34]. Physicians in France found it feasible to adopt the Choosing Wisely^®^ recommendations concerning multiple sclerosis [31]. In USA, primary care providers were ambiguous: Choosing Wisely^®^ items regarding not testing in asymptomatic patients were widely accepted and followed. On the other hand, items concerning testing and treatment in symptomatic patients revealed difficulties in acceptance especially for patients [30]. A large Canadian study revealed that patients are willing to abstain from low-value practices when educated accordingly [33]. Malpractice concerns, patient requests for services and lack of time for shared decision making were the most frequently cited obstacles to reducing low-value practices in the USA [30].
